# Resolving alternative organic crystal structures using density functional theory and NMR chemical shifts†‡

**DOI:** 10.1039/c9sc04964a

**Published:** 2020-02-24

**Authors:** Cory M. Widdifield, James D. Farrell, Jason C. Cole, Judith A. K. Howard, Paul Hodgkinson

**Affiliations:** Department of Chemistry, Oakland University 146 Library Drive Rochester MI 48309-4479 USA; Institute of Physics, Chinese Academy of Sciences Beijing 100190 People's Republic of China; Cambridge Crystallographic Data Centre 12 Union Road Cambridge CB2 1EZ UK; Department of Chemistry, Durham University Stockton Road Durham DH1 3LE UK paul.hodgkinson@durham.ac.uk

## Abstract

Alternative (‘repeat’) determinations of organic crystal structures deposited in the Cambridge Structural Database are analysed to characterise the nature and magnitude of the differences between structure solutions obtained by diffraction methods. Of the 3132 structure pairs considered, over 20% exhibited local structural differences exceeding 0.25 Å. In most cases (about 83%), structural optimisation using density functional theory (DFT) resolved the differences. Many of the cases where distinct and chemically significant structural differences remained after optimisation involved differently positioned hydroxyl groups, with obvious implications for the correct description of hydrogen bonding. ^1^H and ^13^C chemical shifts from solid-state NMR experiments are proposed as an independent methodology in cases where DFT optimisation fails to resolve discrepancies.

## Abstract 2

DFT optimisation often resolves conflicting crystal structure determinations, with NMR shifts helping in cases where optimisation diverges to different structures.

## Graphical abstract

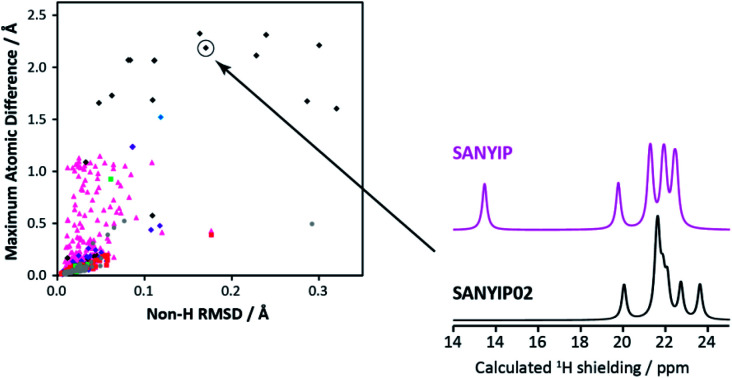

## Introduction

Diffraction techniques are well established for the determination of solid-state structures. Structure solutions for organic crystal structures are typically deposited in the Cambridge Structural Database^1^ (CSD); this repository of crystal structures has been growing approximately exponentially and now contains over one million structures. Beyond their role as repositories of experimental results, such databases have a high impact in several key scientific areas, including: interpreting and understanding intermolecular interactions; developing parameterizations for molecular modelling; and crystallographic structure refinement. It is now common, however, especially for important molecular systems, to find multiple structure determinations for a single solid form (*e.g.* 39 structures are present for the α form of glycine^2^), which complicates the re-use of the structural data. Some of these alternative/repeat determinations differ significantly, and it may be difficult to judge which, if any, of the solutions is “better”. Verification tools, such as checkCIF/PLATON,^3^ may assist the expert crystallographer, but historically many structures have been deposited without structure factors/raw data, limiting the ability to retrospectively validate the diffraction data. Efforts to encourage deposition of the raw data by the crystallographic community are, however, bearing fruit; the Cambridge Crystallographic Data Centre (CCDC) has structure factors or raw intensities for more than 75% of deposits since 2017.

Computational approaches to validating crystal structures based on geometry optimisations using dispersion-corrected density functional theory (DFT) have been described by van de Streek and Neumann; structures were deemed to be unproblematic if the root-mean-square deviation (RMSD) in non-H atomic positions was less than 0.25 Å.^4^ This metric was expressly chosen to avoid picking up differences in H atom positioning, *e.g.* due to differing orientations of methyl groups. Although the primary goal was to show that DFT could successfully reproduce experimental lattice parameters, three of the 225 non-disordered crystal structures, taken from a single issue of *Acta Crystallographica Section E*, were found to contain anomalous features that were ultimately linked to the positioning of hydrogen atoms and/or the modelling of disorder.^4^ This approach was used in follow-on work to validate crystal structures derived from powder X-ray diffraction (XRD) data. There, about 9% (19 out of 215) of structures were deemed to be questionable (using a slightly looser criterion of non-H RMSD > 0.35 Å), reflecting the generally greater potential for ambiguity in solving structures from powder diffraction data, and a higher problem rate in structures not published in International Union of Crystallography (IUCr) journals.^5^ DFT-based geometry optimisation has also been shown to be a valuable tool for refining the position of hydrogen atoms derived from XRD data, reducing the scatter on apparent bond lengths and bringing them in line with values from neutron diffraction studies.^6^

As illustrated below, however, such geometry optimisation starting from different initial structures is not guaranteed to converge to the same structure. Given the importance of correctly positioned hydrogen atoms for describing hydrogen bonding arrangements, independent experimental evidence from a technique that is sensitive to hydrogen atom positioning would be highly desirable. Nuclear magnetic resonance (NMR) spectroscopy is highly sensitive to the local environment about the nuclei studied (for example, effective anisotropic displacement parameters derived from NMR chemical shifts have been estimated to be consistently smaller than those associated with X-ray diffraction^7,8^). “NMR crystallography”^9^ is a rapidly developing field that has recently been recognised by the IUCr in the form of a commission on NMR crystallography.^10^ Having previously observed that ^13^C NMR spectra could discriminate between two alternative structures of terbutaline sulfate present in the CSD^11^ and how ^13^C and ^1^H NMR shifts could be used to validate one of two alternative structures of furosemide,^12^ we wished to determine how frequently alternative structures occurred in the CSD, and whether ^1^H and/or ^13^C chemical shifts could be used routinely to distinguish between such alternative solutions.

We present here results from a comprehensive search of the CSD for alternative structure determinations of organic systems. Structure pairs with potentially significant local differences (at least one atomic displacement >0.25 Å) were geometry optimised using dispersion-corrected projector augmented-wave (PAW) DFT calculations. Where these optimisations failed to resolve the differences, ^1^H and ^13^C magnetic shielding values were calculated, and the likelihood that solid-state NMR experiments would discriminate between the alternative solutions was assessed. The largest fraction of the unresolved structural differences (other than methyl group orientation differences) were found to be associated with hydroxyl group orientations, making them well-suited to distinction *via* solid-state NMR experiments.

## Experimental

The experimental methodology is described in detail in Sections 1–3 of the ESI,‡ and is outlined briefly below. The search for alternative structures was limited to ‘good quality’ (*R* factor ≤10%) structures of typical organic solids (structures containing atoms with an atomic number greater than Cl were excluded). Structures obtained from powder diffraction were included, but represent less than 1% of the structures considered. An initial set of over 200 000 organic structures was grouped according to the leading six characters of their CSD reference code, *i.e.* each group should correspond to the same compound, but potentially different polymorphs. “Plausible” alternative structures were identified by finding pairs within each group having reduced unit cell lattice values within ≤1% (which also avoids comparing structures determined using data acquired at very different pressures). To avoid structural differences associated simply with changing temperature, and cases where one structure was clearly preferred, the experimental temperatures were required to be within 5 K, and the difference in *R* factors to be ≤2%. For each matched pair, the CSD Python application programming interface was used to perform structural overlays, leaving 4238 pairs of structures (although, as discussed in the ESI,‡ only 3132 pairs could be meaningfully compared).

Two parameters were used to quantify the structural differences between alternative structures: the RMSD between non-H atomic positions, but also the maximum displacement between any pair of atoms, including hydrogen atoms. Attempts were made to classify the chemical functionality associated with the maximum displacement (*e.g.*, OH, NH_2_, CH_2_, *etc.*), see ESI‡ for details. Differences between structure pairs were classed as significant if the maximum individual atom–atom displacement exceeded 0.25 Å. As shown in Fig. S3,‡ this criterion selects 658 structures (corresponding to ∼20% of the structure pairs), and is a pragmatic choice reflecting the diminishing returns of running computationally expensive DFT optimisations on structures that are identical within reasonable experimental uncertainties. Mirroring the approach of van de Streek,^4^ up to two rounds of structural relaxation were applied using dispersion-corrected^13^ DFT with the PBE^14^ functional and periodic boundary conditions. In the first optimisation, all atomic positions in the unit cell were relaxed, with the unit cell parameters fixed at the diffraction-derived values. In the minority of structure pairs (∼20%) where this optimisation failed to reduce the maximum displacement to below 0.25 Å, the structures were further relaxed by allowing the unit cell parameters (and all atomic positions) to vary. This was helpful in relatively few cases, and so the figures in this manuscript focus on the results of the fixed-cell optimisation. 113 pairs of structures (out of the original 658) failed to be resolved after the two-step structure relaxation procedure. Note that in a small number (4) of additional cases, optimisation resulted in divergent structures; further investigation would be needed to determine whether these represented potentially distinct polymorphs.

^1^H and ^13^C NMR magnetic shielding values were calculated for the 113 remaining optimised alternative structure pairs, using the gauge-including PAW method,^15,16^ to determine whether ^1^H and/or ^13^C isotropic chemical shifts could reliably distinguish between the alternative structures. A simple automated analysis based on unassigned chemical shifts (ESI,‡ Section 4) showed that shift differences due to methyl group orientations are generally too small to be distinguished. For the smaller subset of pairs with differences in OH positioning, the peaks were assigned to specific chemical sites and the spectral RMSD values for each pair were calculated. These RMSD values were compared to currently accepted ranges for acceptable agreement between experimental and calculated ^1^H and ^13^C chemical shifts (0.33 ± 0.16 ppm for ^1^H, 1.9 ± 0.4 ppm for ^13^C).^17^ The structures were classed as being distinguishable if the RMSD exceeded these thresholds at the 1σ level of confidence. These excellent RMSDs between experimental NMR data and calculations (corresponding to less than 2% of the relevant chemical shift ranges) are obtained, despite the large difference in effective temperature between calculation (0 K) and experiment (ambient temperature). It has previously been shown that low-amplitude libration-type motions have negligible impact on NMR crystallography,^18^ while the dynamics of methyl groups were explicitly taken into account in our comparisons (see ESI‡).

## Results and discussion

**Fig. 1:**
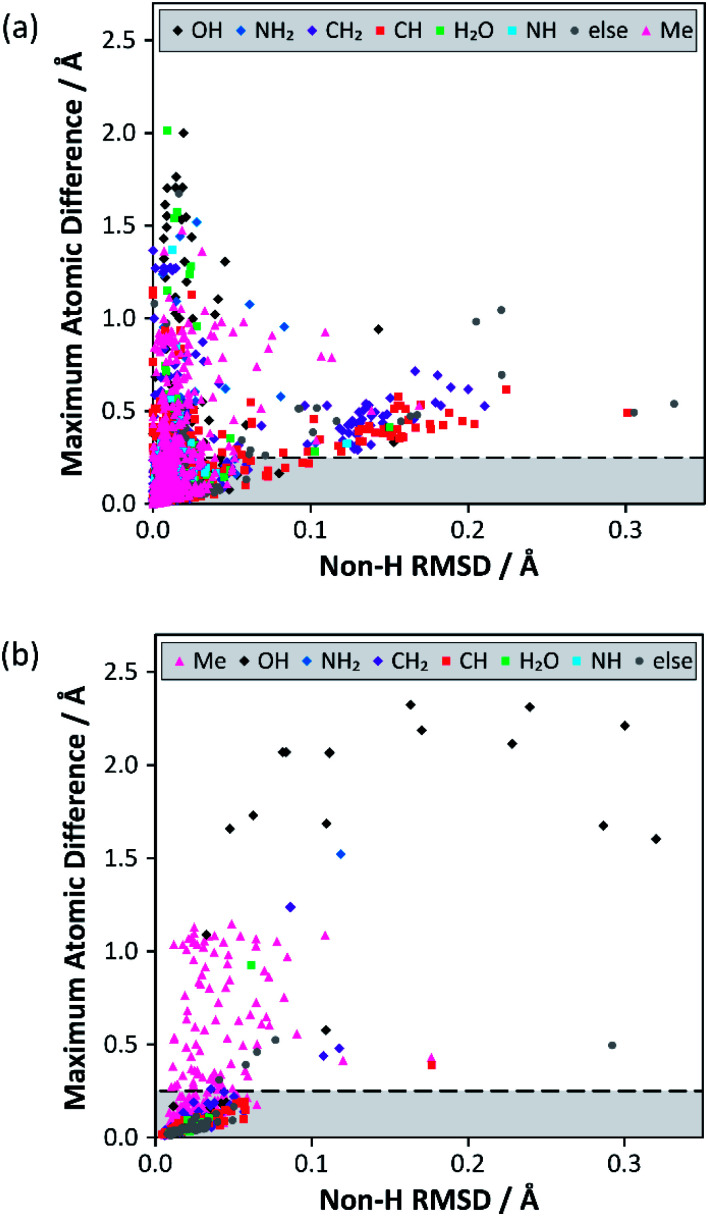
Results from the structure overlay of alternative structure determinations: (a) prior to DFT structural relaxation (excluding 6 outliers) and (b) after DFT optimisation with fixed unit cell parameters (excluding 2 outliers). The horizontal axis specifies the non-H RMSD, while the vertical axis denotes the largest atomic position difference. The horizontal dashed lines at 0.25 Å mark the threshold for structure pairs to be considered sufficiently different to warrant (further) DFT relaxation. The full version of (a), including outliers, can be found in Fig. S12 of the ESI.‡ The ESI also contains simplified versions of (a) broken down by difference type (Fig. S4–S11‡).

Analysis of the alternative structure determinations is summarised in Fig. 1(a), where each datum corresponds to one of 3126 structure pairs (an additional 6 structure pairs were outliers and can be found included in Fig. S12‡). The position of each point is determined by two structural difference metrics: the heavy atom (*i.e.* non-H) RMSD between atomic positions, and the maximum displacement (including H atoms), between any pair of atoms. The colour indicates the associated chemical functionality. This two-dimensional plot is more informative than individual projections of the metrics (see Fig. S3 in the ESI‡). In the vast majority of cases (79%), the atom type associated with the maximum atomic difference was a hydrogen, which is unsurprising given the much larger uncertainties associated with locating H atoms using XRD. Nevertheless, the range of values for this difference is much larger than would be expected purely from random experimental uncertainties. The notion that structural parameters, such as bond lengths, vary between structural determinations by significantly more than the statistical uncertainties indicated by thermal displacement parameters has been made previously.^19^

A large majority of alternative determinations are in good agreement, but there are long tails (as well as individual outliers) in both dimensions. The data points spread out in the direction of the non-H RMSD are largely associated with CH/CH_2_ groups, but most of these structure pairs belong to sucrose conformers (refcode trunk: **SUCROS**) with very slight differences in their ring conformations, all of which resolve upon DFT structural relaxation.

Fig. 1(b) summarises the structural differences after fixed-cell DFT geometry optimisations for 656 structure pairs that initially had a maximum atomic difference of ≥0.25 Å. DFT optimisation almost eliminates the “tail” (noted above) associated with significant differences in overall RMSD. In contrast, a significant fraction of differences associated with H positioning are unresolved, implying that the starting structures have refined to distinct local minima. In these cases, the overall RMSD is often larger after the optimisation, suggesting that the atoms near the largest atomic difference are being moved away from their initial positions to accommodate an alternative placement. Note that only a small number (<1%) of these geometry optimisations would have met the criterion used by van de Streek and Neumann for a potentially suspect structure. That criterion was, however, conservatively chosen to avoid picking up cases associated with H positions. Here, DFT is being used to resolve much smaller discrepancies in local structures that can be potentially picked up by NMR.

Some statistics for the effects of geometry optimisation as a function of diffraction experiment are given in Table S9 of the ESI.‡ These show that the median maximum atomic displacement on optimisation (almost exclusively associated with H atoms) is significantly smaller for neutron data (0.09 Å) compared to that for single-crystal XRD (SC-XRD) data (0.30 Å); this is expected given the much larger uncertainties with locating hydrogen atoms using XRD. On the other hand, there is no significant difference between radiation sources for differences associated with the non-H RMSD axis of Fig. 1. The number of structures derived from powder diffraction data was too small (18) to draw any clear conclusions, although the median RMSD movement on optimisation (0.08 Å) was about a factor of two larger in comparison to SC-XRD studies.

Overall, 113 structure pairs remained sufficiently distinct (*i.e.*, the maximum atomic difference remains ≥0.25 Å) after two rounds of geometry optimisation. These represent pairs of structures in the CSD with very similar *R* factors which cannot be reconciled using DFT. 73 of these cases (Table 1, rightmost column) correspond to alternative methyl group orientations (the cluster with a maximum atomic difference at ∼1 Å in Fig. 1(b) corresponds to methyl groups differing in orientation by 60°). Such behaviour has been observed previously,^20^ but is not chemically interesting, particularly as methyl groups are expected to be dynamic at ambient conditions. These cases do, however, suggest that the data are not unduly biased by redeterminations of problematic structures; nobody would repeat a crystallographic study to check the orientation of a methyl group! Differences in OH group orientation are the second most common type of unresolved structural difference (17 pairs) and are significantly more interesting. As a hydroxyl group can in principle rotate 360° about the R–O(H) bond, it is not surprising that geometry optimisations may diverge to distinct local minima (15% of cases). It has been demonstrated that DFT optimisation is often unsuccessful if bond angles are significantly distorted from their equilibrium values.^6^ In contrast, differences in positioning of NH_2_ groups (180° range of orientation) were resolved by DFT in all but one case. As an example, the structure pair **JISVEM**/**JISVEM01** has NH_2_ groups that differ in orientation by about 90° (maximum atom difference is 1.52 Å), but this converges to a common structure after optimisation. These overall conclusions are expected to be robust with respect to computational parameters, such as the DFT functional; in individual cases where geometry optimisation was applied to a pair of alternate structure solutions, the results were independent of functional.^21^

**Table 1:** Common differences between alternative structure pairs, before and after DFT structural relaxation

Difference type	Initial (%)	Fixed-cell opt. (%)	Full-cell opt. (%)
Methyl	189 (28.7)	88 (65.7)	73 (64.6)
CH	122 (18.5)	1 (0.7)	0 (0)
OH	111 (16.9)	17 (12.7)	17 (15.0)
CH_2_	98 (14.9)	12^a^ (9.0)	11^a^ (9.7)
NH_2_	35 (5.3)	1 (0.7)	1 (0.9)
NH	33 (5.0)	1 (0.7)	1 (0.9)
H_2_O	27 (4.1)	2 (1.5)	0 (0)
NH_3_^+^	18 (2.7)	1 (0.7)	0 (0)
Others	25 (3.8)	11 (8.2)	10 (8.8)
Total	658	134	113

a Value is potentially misleading, as 9 of these pairs involve the same form of one compound (base reference code: **HXMTAM**). Note that percentages may not add to exactly 100% due to rounding.

Potentially, the relative lattice energies determined by dispersion-corrected DFT calculations could be used to predict which of two structures was more likely to be correct; recent computational work showed that differences in lattice energies of stable polymorphs exceeded 7.2 kJ mol^−1^ in only 5% of cases.^22^ Such comparisons, however, require that the geometry optimisations have been converged much more tightly^23^ than was required for this work, and need to be used cautiously when comparing structures with different hydrogen bonding arrangements, given the known deficiencies of DFT in describing hydrogen bonding.^24^ It would thus be pragmatic to use an independent experimental technique, such as solid-state NMR, to discriminate between alternative structures.

**Fig. 2:**
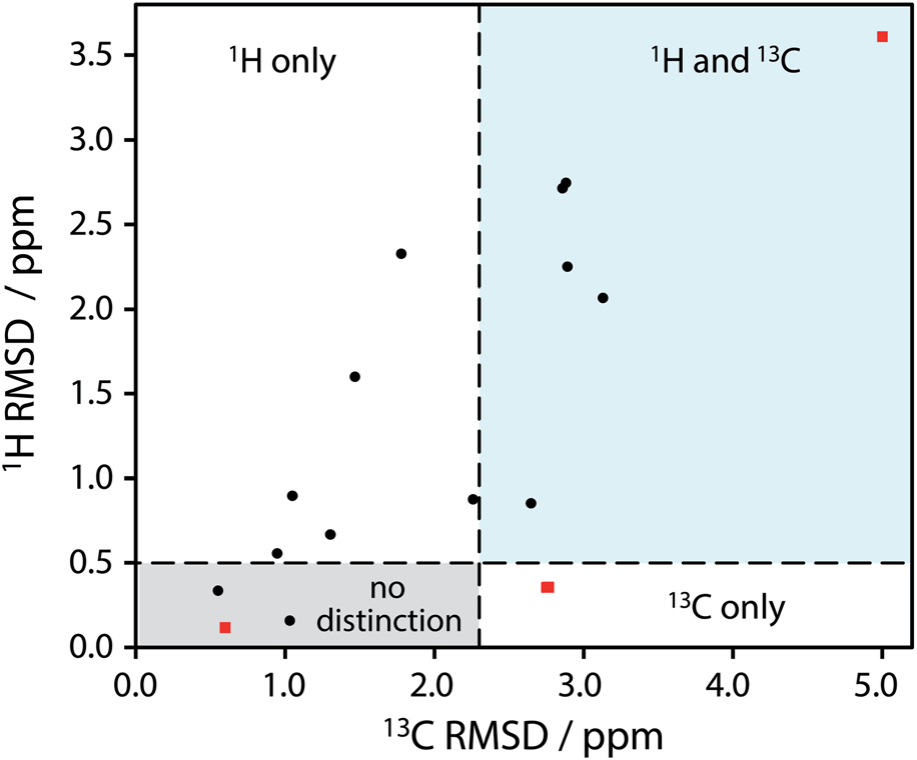
Plot of RMSD between calculated ^1^H and ^13^C isotropic shifts for the 17 structure pairs with OH differences that were unresolved after DFT structural relaxation. The grey region contains structures that are not expected to be distinguishable using either ^1^H or ^13^C NMR experiments, while data in other regions are expected to be distinguishable using ^1^H and/or ^13^C NMR experiments. The horizontal line at 0.5 ppm and the vertical line at 2.3 ppm are based on literature NMR metrics with a 1σ confidence in distinction. Red squares highlight the **SANYIP** (top right), **IPRPOL** (bottom left), and **EDENEH** (bottom right) pairs considered further below. Raw data for this plot are in Table S8 of the ESI.‡

A simple automated analysis based on unassigned chemical shifts (see Section 4 of the ESI‡) showed that differences due to methyl group orientations are expected to be too small to be distinguished using ambient temperature measurements. Given the sensitivity of methyl group chemical shifts to nuclear delocalisation effects^25^ (not accounted for in most quantum chemical calculations), and the lack of structural significance, we have not investigated whether alternative NMR metrics could be used in these cases. In contrast, the documented sensitivity of ^1^H magnetic shielding values to differences in hydrogen bonding^26,27^ implies that differences in OH group positioning should be detectable using ^1^H NMR under fast magic-angle spinning (MAS) and/or standard ^13^C solid-state MAS NMR experiments. More rigorous metrics based on assigned shifts (see ESI‡ for details) were applied to this subset of 17 structure pairs, with the results shown in Fig. 2. (Reviewing the original publications for this subset of the dataset showed no cases where a structure was explicitly being re-determined because of suspected problems with an original structure determination.)

**Fig. 3:**
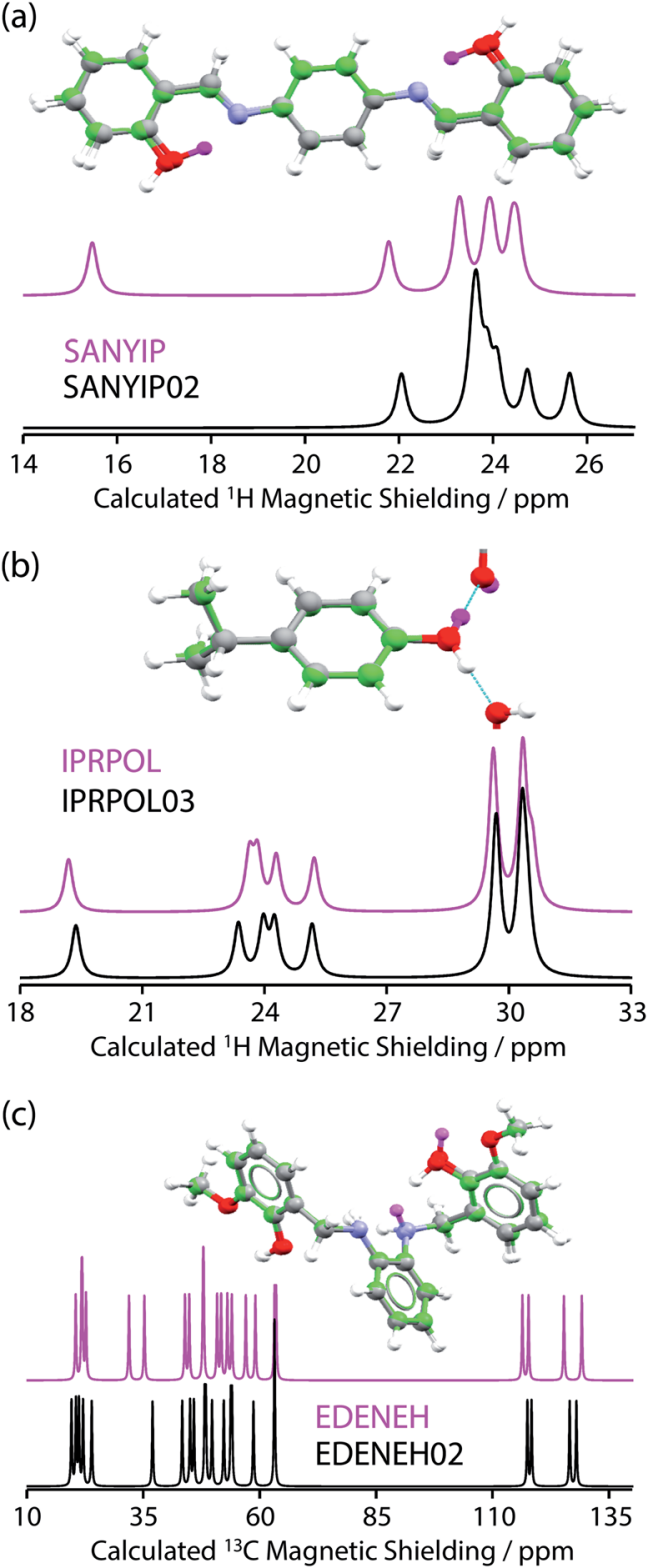
(a, top) Overlaid full-cell geometry-optimised crystal structures of **SANYIP** and **SANYIP02** show minor differences apart from the position of the hydrogen atom of a hydroxyl group (this H atom is highlighted in magenta for **SANYIP**). Calculated ^1^H MAS NMR spectra (a, bottom) show differences in the region around 15.5 ppm. In contrast (b) **IPRPOL** and **IPRPOL03** have very similar calculated ^1^H MAS NMR spectra, despite a different hydroxyl orientation (H atom for **IPRPOL** is indicated in magenta). (c) **EDENEH** and **EDENEH02** are only expected to be distinguishable using ^13^C NMR data, despite significant differences in the positioning of two hydrogen atoms.

From the data in Fig. 2, it is observed that in many cases (14 out of 17 structure pairs) ^1^H and/or ^13^C NMR experiments should discriminate between the alternative pairs of structures. Three of these pairs are considered as representative examples. The pair **SANYIP** and **SANYIP02**, Fig. 3(a), is predicted to be distinguishable using both ^1^H and ^13^C NMR experiments. In **SANYIP**, the H atom of the unique OH is directed toward a nearby nitrogen, forming an intramolecular interaction, while this atom is part of an intermolecular interaction in **SANYIP02** (see also Fig. S14 of the ESI‡). These significant structural differences dramatically alter the computed ^1^H NMR spectra; the calculated shielding of this hydrogen is 15.5 ppm in **SANYIP** and 25.6 ppm in **SANYIP02**, and so ^1^H NMR experiments should distinguish between these two structures with essentially 100% confidence. The structure pair **IPRPOL** and **IPRPOL03**, Fig. 3(b), provides an informative counter-example. Local symmetry means that the two placements do not alter the hydrogen bonding network in a chemically meaningful fashion (this can be seen more clearly in Fig. S15 of the ESI‡). Hence a local probe, such as the NMR chemical shift, is not expected to be able to distinguish these alternative structures with any confidence. In this case, neutron diffraction studies may be necessary if this ambiguity were not acceptable. For the structure pair **EDENEH** and **EDENEH02** (and equivalently, **EDENEH01** and **EDENEH02**) the main structural difference arises from changing an intermolecular O–H⋯N hydrogen bond (as seen in both **EDENEH** or **EDENEH01**) to a moderately weak O–H⋯N intramolecular hydrogen bond in **EDENEH02**. This is shown in greater detail in Fig. S16 of the ESI.‡ Although the structural difference is broadly similar to that observed in the **SANYIP**/**SANYIP02** pair, the ^1^H NMR isotropic shifts are less distinctive than changes in ^13^C isotropic shifts in this case, reinforcing the value of acquiring both ^13^C and ^1^H NMR data. It is also important to note that NMR observables are not limited to isotropic chemical shifts; there are a number of literature examples of using, for example, ^13^C shift anisotropy data to resolve resonances that happen to have very similar isotropic shifts.^28,29^

## Conclusions

We have analysed 3132 instances of repeat structure determinations in the Cambridge Structural Database. While many of the repeat determinations have highly similar structures, the differences are not consistent with a normal distribution of experimental uncertainties, with about 20% of structures showing individual atomic differences greater than 0.25 Å. DFT-based geometry optimisation was used to try to “relax” the structures to a common minimum energy structure. This was successful in over 80% of cases; the relaxed structures are available in the data archive associated with this work. This left 113 structure pairs with unresolved differences. Most of these corresponded to uninteresting differences in methyl group orientation, but the next most common form of unresolved difference corresponded to hydrogen atoms in hydroxyl groups. Based on GIPAW DFT calculations of magnetic shielding, it was shown that ^13^C and/or ^1^H chemical shifts from solid-state NMR experiments should be able to differentiate between alternative positionings of hydroxyl hydrogens in all but a few exceptional cases where the differences are dominated by long-range ordering. Overall this approach of structure distinction *via* solid-state NMR is not burdensome, since the initial step of structural relaxation using periodic DFT is now well established. Such structural relaxation is particularly valuable where structures have been derived from powder diffraction, either as part of the refinement process,^30,31^ or, for example, to confirm the validity of structures which are potentially suspect as suggested by high *R* factors.^32^ Measurement of ^13^C and ^1^H NMR chemical shifts is only needed in the relatively small number of cases where the structural relaxation results in different local minima, and where the differences are structurally significant, *e.g.* if they correspond to different hydrogen bonding arrangements. These conclusions are in line with previous reports of NMR crystallography on individual systems, where NMR has often been used to distinguish between structural solutions involving hydroxyl groups: structures with the same heavy atom positions, but different hydrogen bonding networks;^21^ cases where a single OH group has a different orientation;^11,12^ or distinguishing between solutions from powder XRD studies, where the orientation of OH groups is often ambiguous.^33,34^

## Conflicts of interest

There are no conflicts to declare.

## Supplementary Material

SC-011-C9SC04964A-s001
