# Supplementary material for: Resolving alternative organic crystal structures using density functional theory and NMR chemical shifts
Source: Chem Sci. 2020 Feb 24;11(11):2987–92. doi: 10.1039/c9sc04964a (PMC8157514; doi:10.1039/c9sc04964a)
Supplement: SC-011-C9SC04964A-s001 [file SC-011-C9SC04964A-s001.pdf]

Electronic Supplementary Material (ESI) for Chemical Science.
This journal is © The Royal Society of Chemistry 2020

Resolving Alternative Organic Crystal Structures using Density Functional Theory and NMR Chemical Shifts: Supplementary Information

1. Identification of Repeat Organic Structures in the Cambridge Structural Database (CSD)

Using the ConQuest software[1] developed by the Cambridge Crystallographic Data Centre (CCDC), the following parameters were used to search for structures in the CSD (v.5.36, November 2014 + 1 update): (1) both carbon and hydrogen atoms are found in the same molecule; (2) atomic number restricted to being less than or equal to 17 (i.e., $Z \leq 17$); (3) crystallographic R-factor ≤ 10%; (4) structures are not disordered; (5) structures are not flagged as containing errors; (6) are not polymeric; (7) 3D coordinates are known. This search identified **204 710** crystal structures.

The crystal structures identified above were retrieved from the CSD, and saved as individual CIF-format files. A comma-separated file was generated containing the following information about each of the structures: (i) CSD refcode; (ii) R-factor; (iii) space group; (iv) Z value; (v) Z' value; (vi) experimental measurement temperature; (vii) a, b, and c unit cell values; (viii) α, β, and γ unit cell angles; (ix) unit cell volume; (x) reduced unit cell dimensions and reduced cell volume; (xi) publication year. This file is part of the data archive associated with this manuscript (**Search-Dec15.csv**).

A Python script was used to parse over the above crystal structures (**filter1.py** in the data archive). The script first identifies which of the organic crystal structures are potentially repeat structure determinations by looking for exact matches in the first 6 characters of their associated CSD refcodes.[i] The initial set of **204 710** crystal structures was reduced by this matching to **21 047** structures. A listing of the structures selected thus far is in the data archive (**1stpass_match.txt**).

At this point, the selected structures may contain polymorphs rather than only repeat determinations of the same polymorphic form. Hence, the **21 047** structures were subjected to a more detailed comparison as part of the **filter1.py** script. Within groups of structures sharing a common six character CSD refcode trunk, the following parameters were compared over all possible pairwise combinations of structures (**28 900** structure pairs): (i) space group number; (ii) experimental measurement temperature; (iii) R-factor; and (iv) reduced unit cell lengths (3 comparisons). If a given pair of structures had identical space group numbers,[ii] and all remaining parameters agreed to within selected tolerances, this pair of structures was counted as a match, given an ID label, and retained for subsequent analysis. Different tolerances for (ii)–(iv) were considered, as summarized in Tables S1 – S3 and Figure S1 below.

Considering the variation in the number of matched structure pairs as a function of the various tolerances, it was decided that tolerances of 5 K, 2%, and 1% for ΔT, ΔR and Δa, respectively, represented a balance allowing for a selection of structures that: (i) should represent alternative structure determinations under similar conditions; (ii) are equally plausible (a structure with a higher R factor would tend to be dismissed in favor of a structure with a lower R); and (iii) present a tractable situation, bearing in mind that future analysis steps will become increasingly resource intensive (*vide infra*). Using the above tolerances, **4 238** structure pairs, corresponding to **6 664** unique structures, were selected for analysis.[iii] A list of the structures selected at this point is in the data archive (**2ndpass_match.txt**).

---

[i] A "refcode" is a unique alphanumeric string that is associated with each CSD entry. All refcodes begin with 6 alphabetic characters, with two numeric characters being appended in some instances.

[ii] **22 378** of the **28 900** pairs of structures were found to have identical space group numbers.

[iii] Technically, **4 240** pairs were matched according to the tolerances specified above. However, it was subsequently discovered that 2 structure pairs (CSD refcodes: FEWNOK/FEWNOK10; SEMLAB/SEMLAB02) contained atoms with $Z > 17$ and hence should not have been selected according to the criteria specified above.

Table S1. Number of structure pairs accepted as alternative determinations as functions of parameter tolerances (with $\Delta T \leq 2$ K)[a]

Δa / % ΔR / %	**0.00**	**0.25**	**0.50**	**1.00**	**1.50**	**2.00**	**2.50**	**3.00**
**0.0**	852	984	994	999	1006	1009	1010	1012
**0.5**	1163	1839	2057	2190	2278	2350	2401	2444
**1.0**	1283	2310	2656	2860	3014	3125	3207	3267
**1.5**	1368	2642	3091	3367	3564	3700	3806	3881
**2.0**	1420	2869	3408	3741	3963	4114	4237	4320
**3.0**	1480	3166	3800	4189	4436	4599	4733	4825
**4.0**	1512	3327	4028	4454	4719	4898	5043	5142
**5.0**	1526	3420	4160	4614	4885	5073	5226	5326

[a] Column headings represent the tolerances when comparing *each* of the *a*, *b*, and *c* values for the corresponding reduced unit cells of a structure pair. Structure pairs must be within these tolerances for all three reduced unit cell length values to be accepted. Row headings represent the tolerance when comparing *R*-factors between structure pairs.

Table S2. Number of structure pairs accepted as alternative determinations as functions of parameter tolerances (with $\Delta T \leq 5$ K)[a]

Δa / % ΔR / %	**0.00**	**0.25**	**0.50**	**1.00**	**1.50**	**2.00**	**2.50**	**3.00**
**0.0**	860	993	1003	1008	1015	1018	1019	1021
**0.5**	1182	1990	2249	2390	2487	2563	2616	2662
**1.0**	1309	2251	2964	3187	3355	3472	3558	3624
**1.5**	1398	2943	3479	3785	3998	4142	4255	4336
**2.0**	1451	3215	3866	**4240**	4481	4641	4773	4862
**3.0**	1511	3559	4332	4771	5039	5213	5359	5458
**4.0**	1543	3740	4593	5075	5365	5555	5713	5819
**5.0**	1557	3844	4747	5261	5557	5757	5923	6030

[a] Column headings represent the tolerances when comparing *each* of the *a*, *b*, and *c* values for the corresponding reduced unit cells of a structure pair. Structure pairs must be within these tolerances for all three reduced unit cell length values to be accepted. Row headings represent the tolerance when comparing *R*-factors between structure pairs. The entry in **bolded red** indicates the combination of parameter tolerances selected for the next phase of the study.

Table S3. Number of structure pairs accepted as alternative determinations as functions of parameter tolerances (with $\Delta T \leq 10$ K)[a]

Δa / % ΔR / %	**0.00**	**0.25**	**0.50**	**1.00**	**1.50**	**2.00**	**2.50**	**3.00**
**0.0**	861	998	1008	1013	1020	1023	1024	1026
**0.5**	1187	2084	2367	2518	2618	2695	2749	2795
**1.0**	1317	2684	3138	3373	3546	3664	3752	3818
**1.5**	1407	3109	3692	4015	4234	4380	4496	4578
**2.0**	1460	3402	4111	4505	4752	4916	5051	5141
**3.0**	1520	3766	4606	5066	5340	5518	5668	5769
**4.0**	1552	3955	4881	5385	5681	5876	6039	6147
**5.0**	1567	4073	5052	5589	5891	6096	6267	6376

[a] Column headings represent the tolerances when comparing *each* of the *a*, *b*, and *c* values for the corresponding reduced unit cells of a structure pair. Structure pairs must be within these tolerances for all three reduced unit cell length values to be accepted. Row headings represent the tolerance when comparing *R*-factors between structure pairs.

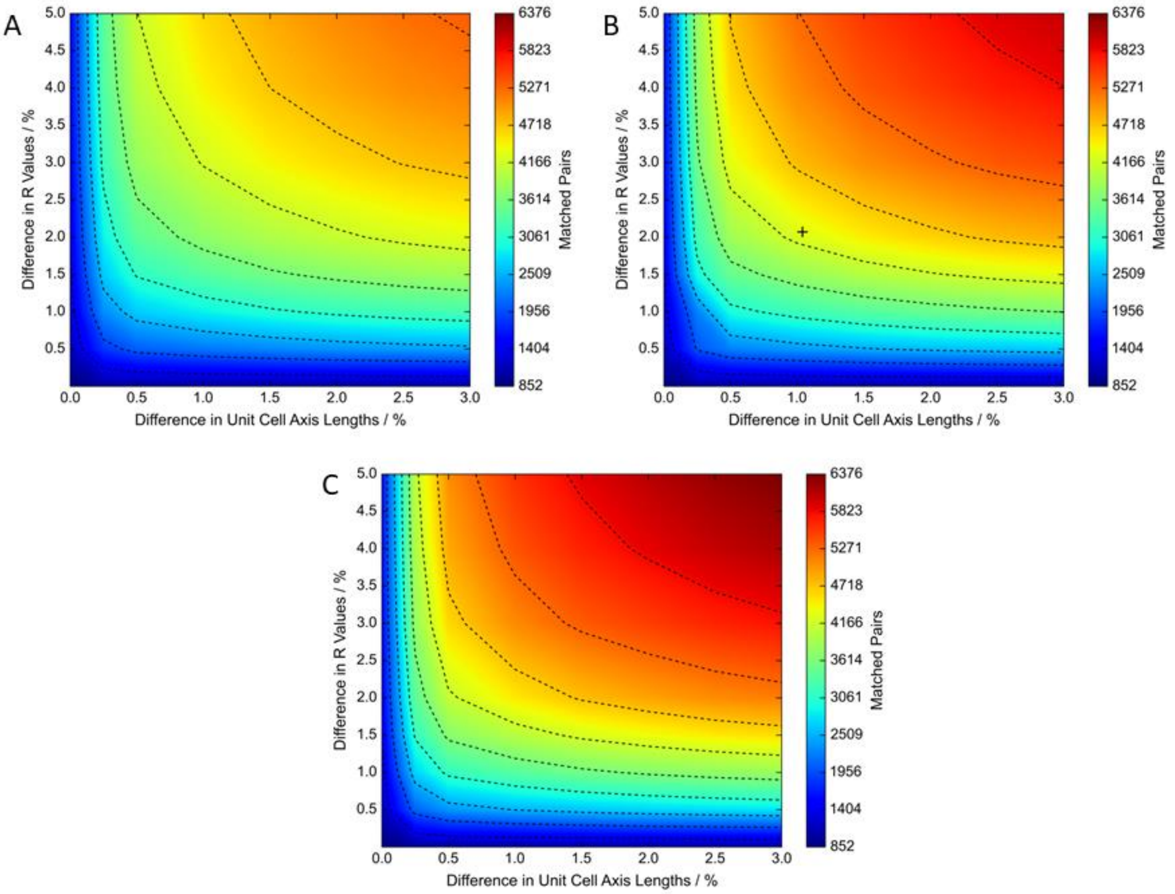

Figure S1. Variation in the number of matched structure pairs as a function of the *R* factor tolerance (vertical axes) and the tolerance in the reduced unit cell axis lengths (horizontal axes) for three Δ*T* values (Δ*T* being the tolerance in experimental temperatures). **A**: Δ*T* = 2 K; **B**: Δ*T* = 5 K; **C**: Δ*T* = 10 K. Each plot was generated by performing discrete calculations at 64 points in the parameter space (data in Tables S1 – S3), followed by interpolation to create a continuous plot. The point on plot **B** indicated with a cross specifies the tolerances used to select the structure pairs that were used in subsequent RMSD calculations (4240 matched pairs).

2. Quantification of Alternative Organic Structure Determination Differences/Similarities

For each of the **4 238** pairs of structures identified above, an overlay of both the asymmetric unit(s) and unit cell contents were performed (the latter when possible) to quantify differences in structure for each pair. The overlay process was semi-automated using the CSD Python API (versions 0.7 and 1.0), with API calls being made using variations of the Python script **overlay.py** (included in data archive). Due to the developmental nature of the CSD Python APIs, it was not possible to cleanly execute this Python script across all structure pairs, and in a significant minority of cases, it was not possible to compare/overlay the structures using an automated approach. In these cases, RMSD information was determined manually using the Mercury software[2] (version 3.7 or 3.8).

While the most significant results derived from the structure pair comparisons are discussed in the main manuscript, here we briefly discuss other details. To produce quantitative results from the comparisons, it was required that both structures: (i) contained the same number of atoms; (ii) represented the same chemical structure; (iii) had fully-specified coordinates for all atoms; and (iv) had

atomic occupancies always equal to 1 (i.e., no disorder). After applying these criteria, **933** pairs of structures were rejected due to missing one or more atoms, while **173** structure pairs were rejected due to either possessing disorder, being otherwise ambiguous in the placement of at least one atom in the structure, or were found to not correspond to the same chemical structure (the "difference type" for these cases is given as "undefined" in the spreadsheet described below). While some of these problems could potentially be individually addressed (e.g. adding missing hydrogen atoms), the loss of less than 25% of the comparisons is unlikely to distort the statistics. Removing these pairs of structures from consideration, **3 132** structure pairs remained that we could overlay to calculate the heavy atom (i.e., non-H atom) RMSD, the all-atom RMSD, and the largest local difference. After determining the heavy atom RMSD, the pair of atoms (one atom from each structure) with the largest local difference was determined using outputs from the CSD Python APIs and the above-mentioned Python script. This script also tried classify this pair of atoms according to familiar chemical motifs.

This classification into "difference types" was accomplished using the CSD API to output (irrespective of the literal atomic labels) the atom types, and number of each atom type, that were bonded to a given heavy atom. For many comparisons, assigning differences to a type was then relatively straightforward (e.g., OH groups); however, for other difference types, further explanation is required. For example, to compute the largest difference between hydrogen atoms associated with two methyl groups (one group equals three H atoms from each of the structure pairs), all hydrogen atomic coordinates were first expanded into six 3D arrays. Three vectors (each with three elements) were then formed by calculating the magnitude of the difference between all possible permutations of the hydrogen atoms under consideration. For example, the first vector would be composed of three elements, with the first element being the magnitude between the "first" H atom in the "first" structure and the "first" H atom in the "second" structure; the second element being the magnitude between the "first" H atom the "first" structure and the "second" H atom in the "second" structure and the third element being the magnitude between the "first" H atom the "first" structure and the "third" H atom in the "second" structure. Overall, this process would produce nine elements for the two methyl groups. Using the smallest value, one atom from each structure was notionally removed, and the process was iterated to leave one pair of H atoms (one from each structure) corresponding to the maximum distance between H atoms for these two methyl groups. Summaries of the results from the overlay process for each pair can be found in the Excel spreadsheet in the data archive (**overlay_results_and_DFT_summaries.xlsx**), an extract of which is provided in Figure S2. Figure S3 shows the number of structure pairs possessing specified amounts of local and overall structural differences, while the most common types of structure pair differences are summarized in Table S4.

PAIR_ID	XTAL1	XTAL2	NEUTRON?	POWDER?	ZVALUE	ZVALUE_RMSD / Å	Z_PRIME	Z	Z_RMSD / Å	RMSDH_int / Å	MAX_ATOM1	MAX_ATOM2	DIST / Å	DIS_TYPE
**0001**	ABAMEX01	ABAMEX02	neither	neither	4	0.00871	1.0	2	0.00792	0.05892	H14	H7	0.2339	Me
**0003**	ABEFOE	ABEFOE01	neither	neither	2	0.00000	0.5	2	0.00000	0.00000	C1	C1	0.0000	None
**0004**	ABEGAR	ABEGAR01	neither	neither	4	0.00000	1.0	2	0.00000	0.00000	C1	C1	0.0000	None
**0005**	ABEGIZ	ABEGIZ01	neither	neither	2	0.00000	1.0	2	0.00000	0.00000	O1	O1	0.0000	None
**0009**	ABINOS	ABINOS01	ABINOS01	neither	4	0.01845	1.0	2	0.01687	0.17951	H9	H8	0.5662	OH
**0010**	ABULIT06	ABULIT07	neither	neither	8	0.02212	1.0	2	0.01124	0.03388	H1	H1	0.1122	OH
**0011**	ABUPUI	ABUPUI01	neither	neither	2	0.00542	1.0	2	0.00542	0.02948	H7	H7	0.0753	Me
**0012**	ACANIL	ACANIL03	ACANIL03	neither	8	0.00797	1.0	2	0.00660	0.06827	H7	H7	0.1939	Me
**0013**	ACEMID03	ACEMID05	ACEMID03	neither	18	0.01044	1.0	2	0.00589	0.09883	H2	H2	0.1453	NH2
**0014**	ACETIM	ACETIM01	neither	neither	2	0.00980	0.5	2	0.00980	0.03783	H7	H1	0.1020	CH2
**0016**	ACMEPT	ACMEPT10	neither	neither	4	0.00000	1.0	2	0.00000	0.00000	C1	C1	0.0000	None
**0018**	ACPHQU	ACPHQU10	neither	neither	4	0.00608	1.0	2	0.00578	0.04662	H5	H5	0.2127	CH
**0019**	ACSALA02	ACSALA09	both	neither	4	0.01317	1.0	2	0.01232	0.03340	H7	H7	0.1104	Me
**0020**	ACUGUA	ACUGUA01	neither	neither	2	0.01671	0.5	2	0.01671	0.02069	H4	H4B	0.0558	CH
**0021**	ACUVAX	ACUVAX01	neither	neither	4	0.00000	1.0	2	0.00000	0.00000	O1	O1	0.0000	None
**0025**	ADENDH	ADENDH03	neither	neither	4	0.00517	0.5	6	0.00561	0.05345	H1	H7	0.1544	NH
**0027**	ADEQUW	ADEQUW01	neither	neither	1	0.03911	0.5	10	0.01595	0.05065	H17	H16	0.2136	H2O
**0028**	ADERIL	ADERIL01	neither	neither	4	0.01365	2.0	4	0.01365	0.24082	H6	H2	0.8522	Me
**0029**	ADGALA01	ADGALA10	neither	neither	4	0.01974	1.0	2	0.01929	0.67729	H9	H11	2.0001	OH
**0030**	ADICOF	ADICOF01	neither	neither	4	0.01806	2.0	4	0.01806	0.05379	H17	H3	0.2458	CH
**0032**	ADIPAC04	ADIPAC11	neither	neither	2	0.00192	0.5	2	0.00192	0.02113	H3	H3	0.0408	CH2
**0033**	ADIPAC06	ADIPAC18	neither	neither	2	0.01205	0.5	2	0.01205	0.09186	H5B	H1	0.2874	OH
**0034**	ADIPAC08	ADIPAC09	neither	neither	2	0.00425	0.5	2	0.00425	0.02290	H1B	H5	0.0729	OH
**0035**	ADIPAC08	ADIPAC14	neither	neither	2	0.00319	0.5	2	0.00319	0.03009	H1	H5	0.0944	OH
**0036**	ADIPAC09	ADIPAC14	neither	neither	2	0.00569	0.5	2	0.00569	0.01416	H5B	H5	0.0418	OH
**0037**	ADTALO01	ADTALO10	neither	neither	4	0.01171	1.0	2	0.00760	0.07718	H12	H12	0.2338	OH
**0039**	AFANAW	AFANAW01	neither	neither	4	0.01390	1.0	2	0.00749	0.03747	H9	H9	0.1115	OH
**0040**	AFIFAX	AFIFAX01	neither	neither	4	0.01062	1.0	2	0.00983	0.04220	H18	H21	0.1180	CH2
**0041**	AGLYSL	AGLYSL01	AGLYSL01	neither	4	0.01550	1.0	6	0.01494	0.16837	H7	H7	0.3878	OH
**0042**	AHARFU	AHARFU01	neither	neither	8	0.04003	2.0	4	0.04119	0.13184	H6	H18	1.1044	OH
**0044**	AHEMAB	AHEMAB02	neither	neither	2	0.00960	0.5	2	0.00960	0.01042	H2B	H2	0.0169	CH

Figure S2. Screenshot capture of the data archive file: **overlay_results_and_DFT_summaries.xslx,** which summarizes the outputs produced by the Python script: **overlay.py**. Each row corresponds to the structural comparison of one pair of alternative structures. The column headings are: **PAIR_ID**, the unique structure pair identification number assigned by the Python script **filter1.py**; **XTAL1** and **XTAL2**, CSD refcodes associated with the "first" and "second" structures of the pair, respectively; **NEUTRON?**, identifies structures obtained using neutron diffraction data; **POWDER?**, identifies structures obtained from powder diffraction data; **ZVALUE**, number of "components" (components can be molecules or ions) in the unit cell; **ZVALUE_RMSD**, heavy atom (non-H) RMSD value when comparing the components contained within the unit cells of the pair; **Z_PRIME**, number of components in each asymmetric unit; **Z_RMSD**, heavy atom (non-H) RMSD value when comparing (typically) 2 times the number of components specified by Z_PRIME (this value was used in various plots, *vide infra*); **RMSDH_int**, an estimate of the all-atom RMSD within the overlay frame of reference generated when determining Z_RMSD; **MAX_ATOM1** and **MAX_ATOM2**, atom labels of the pair of corresponding atoms making the largest contribution to RMSDH_int; **DIST**, distance (in Å) between MAX_ATOM1 and MAX_ATOM2; **DIS_TYPE**, classification of maximum atomic difference.

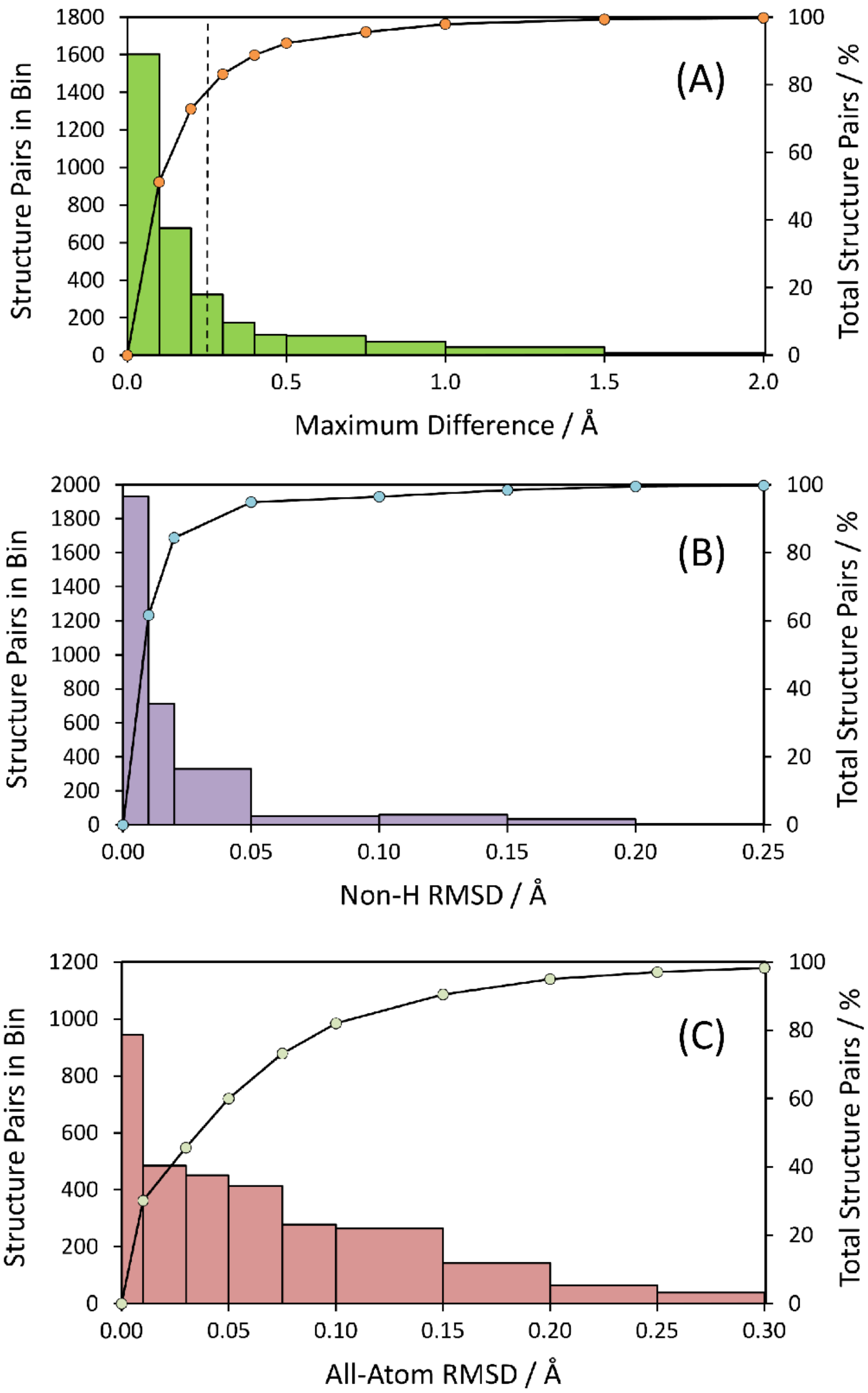

Figure S3. Histograms showing on the primary vertical axis the number of structure pairs possessing (A) a maximum (local) difference, (B) a heavy-atom (non-H) RMSD, and (C) an all-atom RMSD within the values specified by the horizontal axis. The secondary vertical axis indicates the cumulative total number of structure pairs, expressed as a percentage of the **3132** structure pairs being considered at this point. **658** structure pairs having a maximum (local) difference of ≥ 0.25 Å (indicated by the vertical dashed line in A) were selected for a structural relaxation using dispersion-corrected DFT.

Table S4. Most common types of structure pair differences prior to structure optimization

Difference Type	Number of Pairs	Percentage
CH	679	21.7
Me	571	18.2
CH_2	353	11.3
OH	346	11.0
NH	167	5.3
NH_3^+	144	4.6
NH_2	139	4.5
H_2O	93	3.0
None[a]	506	16.2
All others[b]	134	4.3

[a] A pair of structures was assigned a difference type of 'None' if the largest atomic contribution to the non-H RMSD was ≤ 0.001 Å.
[b] Difference types grouped into the 'All others' category (number of pairs in parentheses) include: NH_4^+ (8); Cl^- (8); MeOH (1); BF_4^- (5); $AlCl_4^-$ (1); NO_2 (5); AlH (2); NMe_3 (1); CO (12); CN (4); CNC (2); BH (4); PH (2); SH (5); CF_3 (2); CCl (8); ring (3); NO (1); H_3O^+ (1); rotamer (1); SO_2 (2); CF (2); H-transfer (2); ClO_4^- (2); CS (4); other non-specified differences (46).

While Figure 1a in the main manuscript illustrates the maximum local difference versus the non-H RMSD for nearly all the 3132 structure pairs in an efficient manner, certain details of the plot are obscured by its data-rich nature. Figures S4 – S11 are plots of the maximum local differences versus the non-H RMSD values broken down by the most common types of local differences.

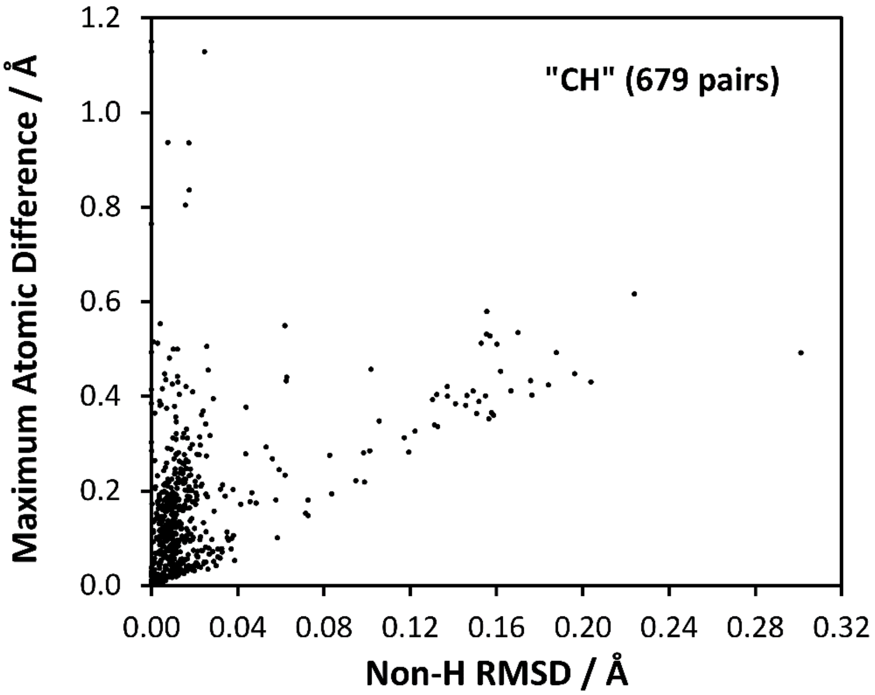

Figure S4. Maximum atomic difference vs. the heavy atom (i.e., non-H) RMSD for the 679 pairs of structures where the maximum atomic difference was classified as being due to a CH moiety.

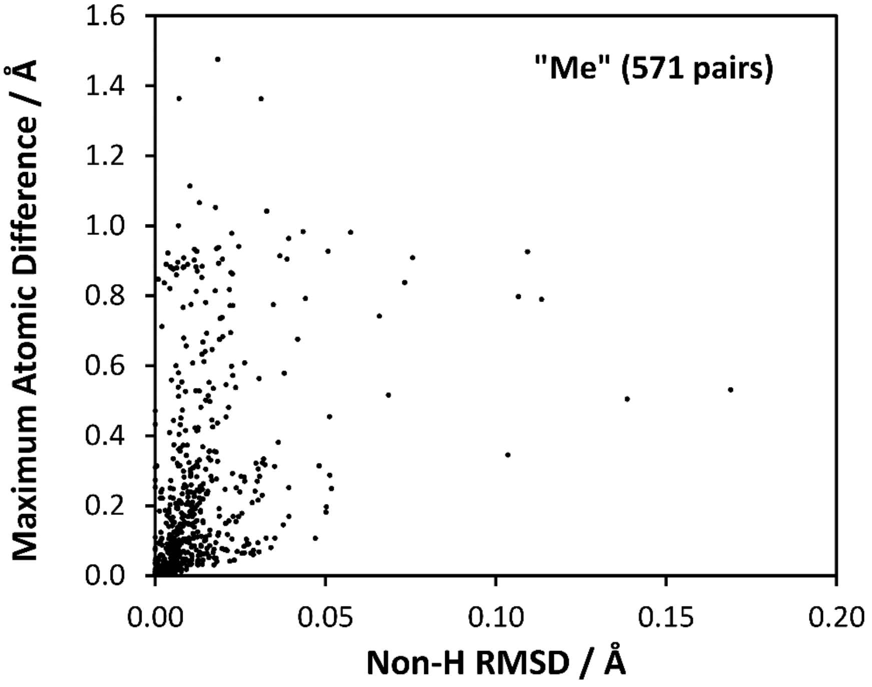

Figure S5. Maximum atomic difference vs. the heavy atom (i.e., non-H) RMSD for the 571 pairs of structures where the maximum atomic difference was classified as being due to a methyl (Me) moiety.

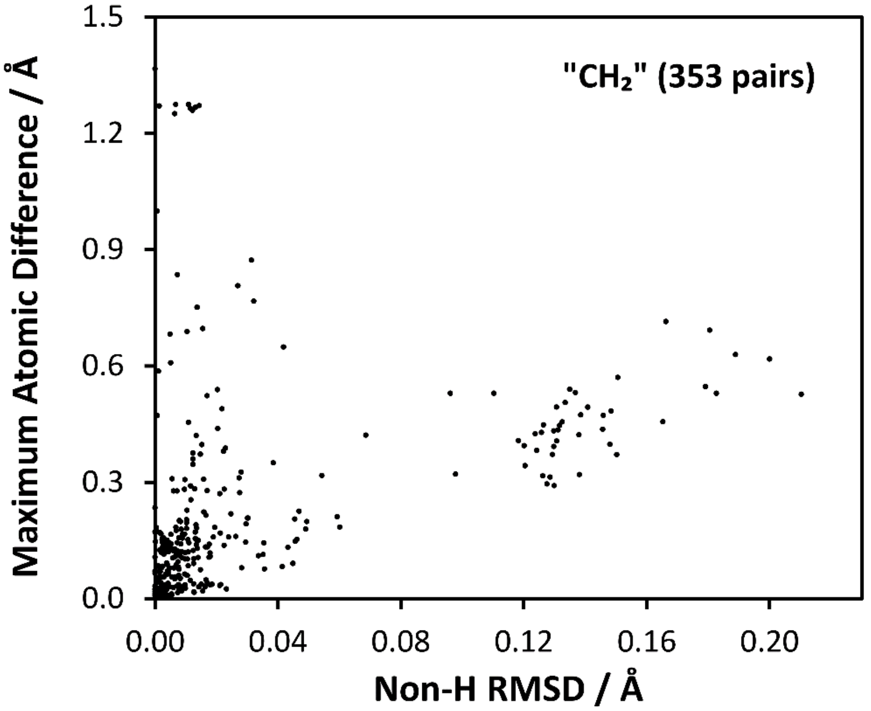

Figure S6. Maximum atomic difference vs. the heavy atom (i.e., non-H) RMSD for the 353 pairs of structures where the maximum atomic difference was classified as being due to a methylene (CH_2) moiety.

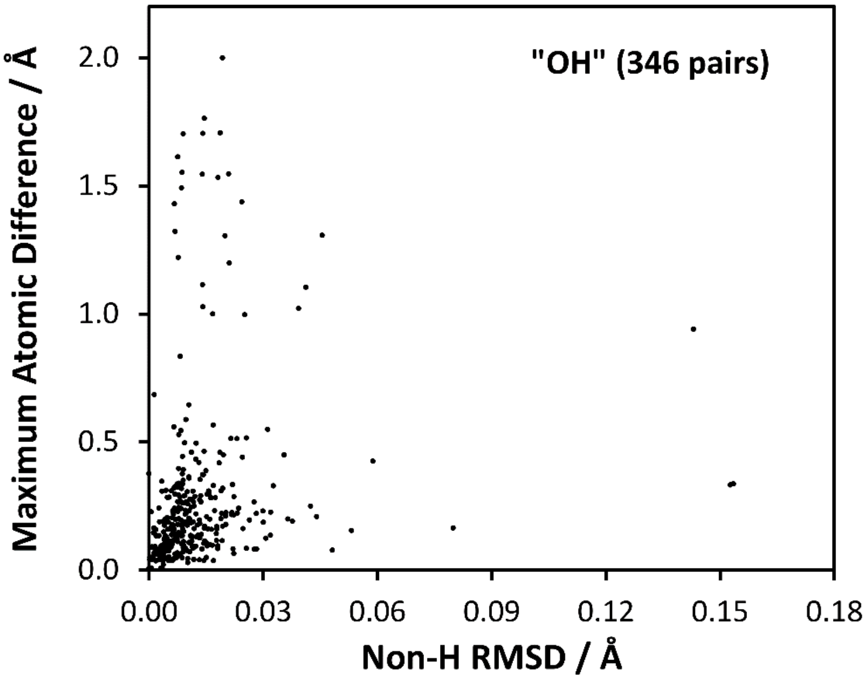

Figure S7. Maximum atomic difference vs. the heavy atom (i.e., non-H) RMSD for the 346 pairs of structures where the maximum atomic difference was classified as being due to a hydroxyl (OH) moiety.

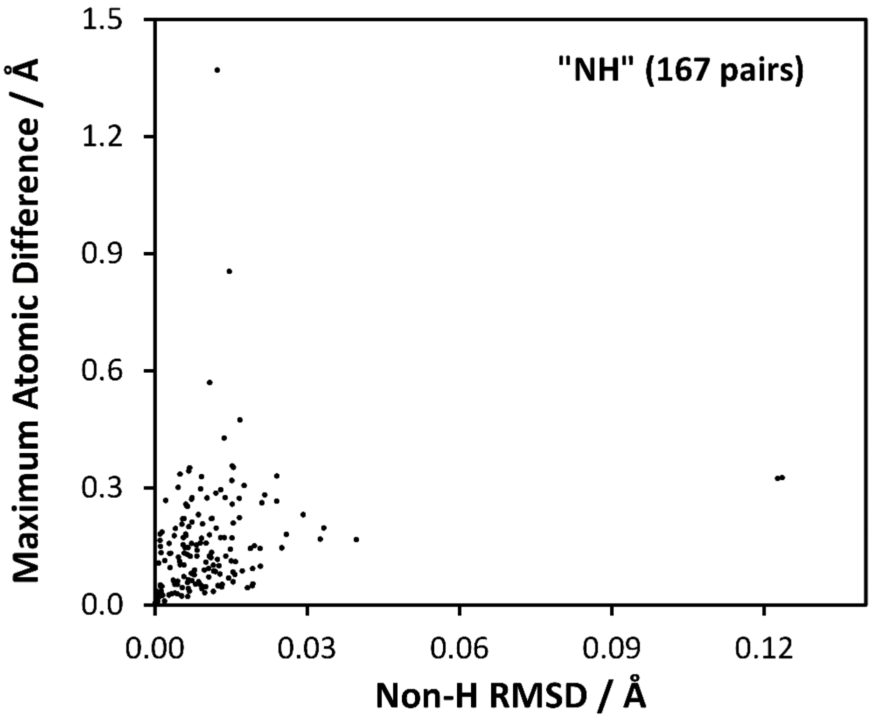

Figure S8. Maximum atomic difference vs. the heavy atom (i.e., non-H) RMSD for the 167 pairs of structures where the maximum atomic difference was classified as being due to an NH moiety.

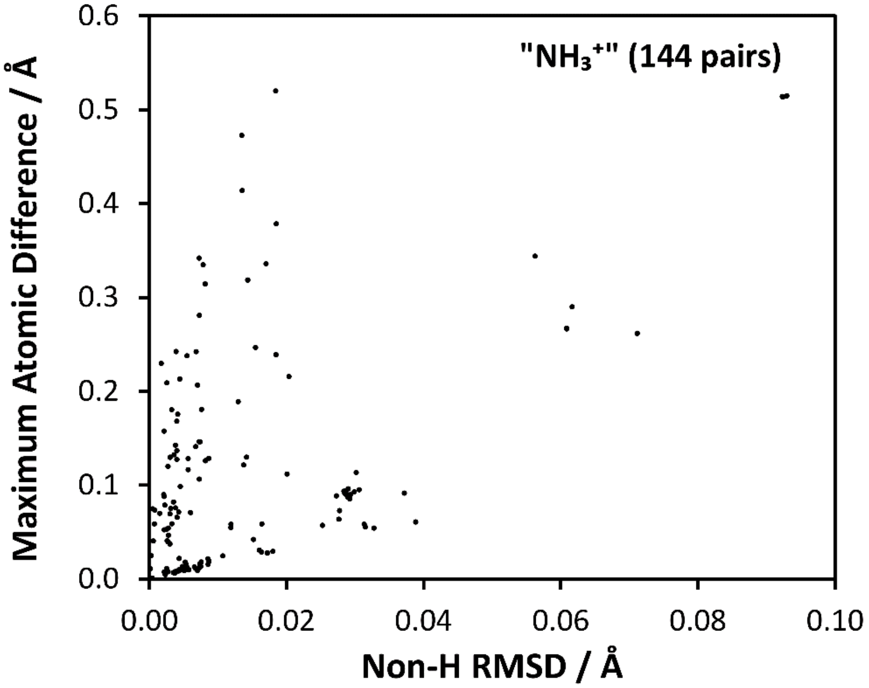

Figure S9. Maximum atomic difference vs. the heavy atom (i.e., non-H) RMSD for the 144 pairs of structures where the maximum atomic difference was classified as being due to an NH_3^+ moiety.

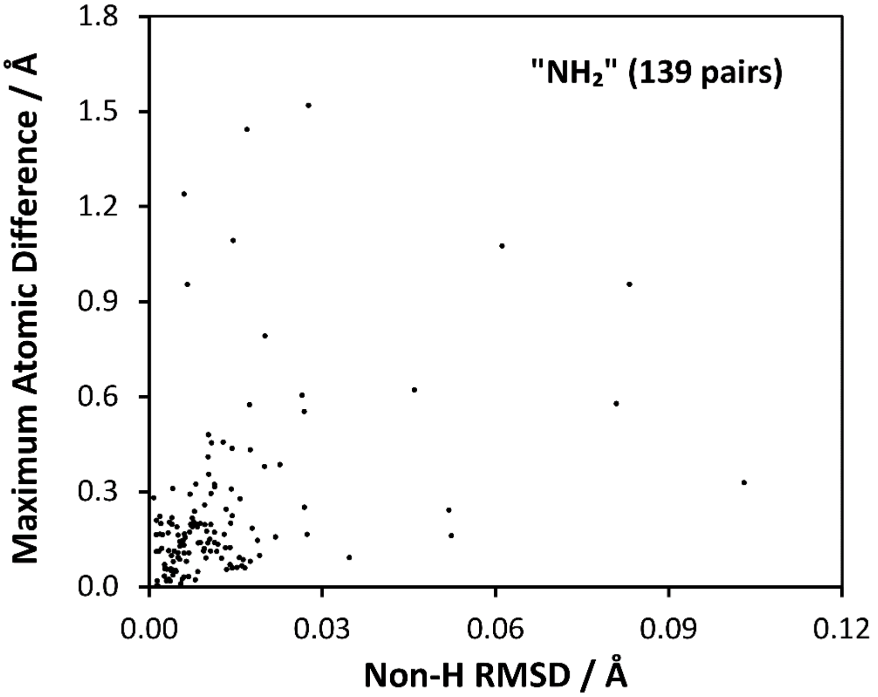

Figure S10. Maximum atomic difference vs. the heavy atom (i.e., non-H) RMSD for the 139 pairs of structures where the maximum atomic difference was classified as being due to an NH_2 moiety.

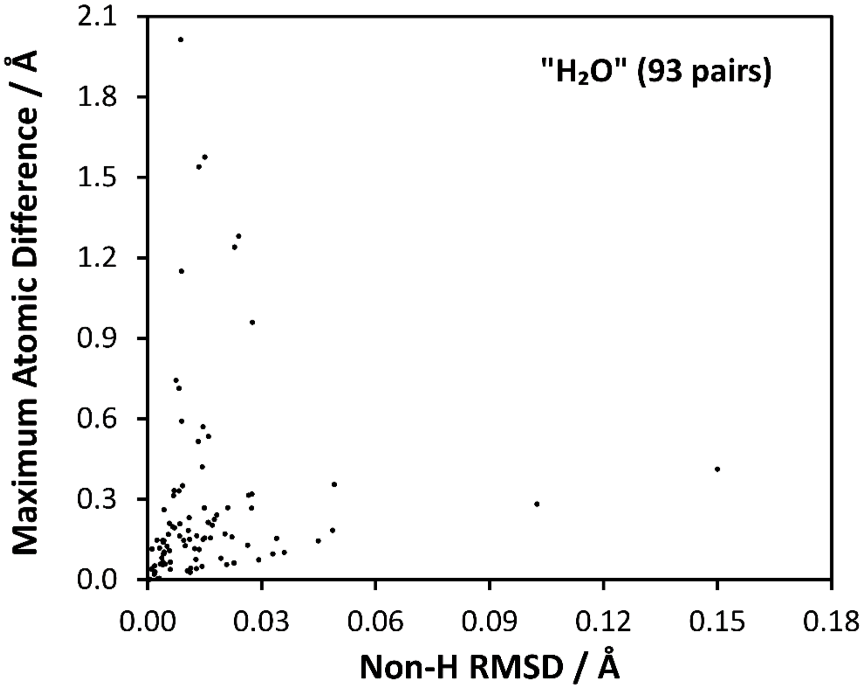

Figure S11. Maximum atomic difference vs. the heavy atom (i.e., non-H) RMSD for the 93 pairs of structures where the maximum atomic difference was classified as being due to a water (H_2O) moiety.

Extremely large local difference/RMSD data points (i.e., outliers) were not included in Figure 1a in the main manuscript. Figure S12 presents the same data as in Figure 1a, but includes the 6 outliers (out of 3132). These outliers involve cases such as asymmetric rings which differ in orientation between alternative structure solutions (e.g. GOKREE/GOKREE01 and ZITZUX/ZITZUX01), and other groups with alternative positions e.g., sulphonamide group orientation (e.g. QQQAUG01/QQQUAG02). As these are individual cases, which may be complicated by disorder, they were not investigated further.

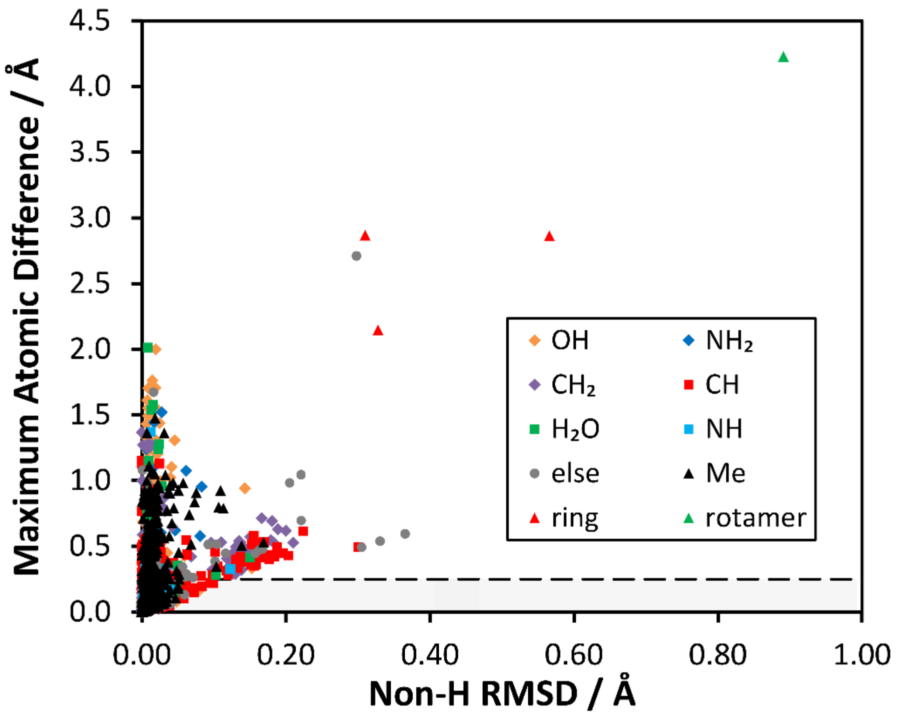

Figure S12. Full results from the structural overlay of alternative structure determinations found in the CSD prior to DFT structural relaxation. The horizontal axis specifies the non-H RMSD value, while the vertical axis denotes the maximum atomic separation for any pair of corresponding atoms. The horizontal dashed line at 0.25 Å indicates the boundary between structure pairs considered sufficiently different to warrant further analysis, and those which were not.

3. Selection of Structure Pairs with Significant Differences, and Attempting to Resolve These Differences Using Dispersion-Corrected DFT Structural Relaxations

After the structure overlay and difference type classification, dispersion-corrected DFT structural relaxations ("geometry optimizations") were used to see if the pairs of structures relaxed to similar structural/energetic minima. As noted above, structure pairs whose maximum atom-atom difference was ≤ 0.25 Å were deemed to be sufficiently similar and were not considered further, leaving **658** structure pairs to be investigated.

To perform the structure relaxations, the Cambridge Serial Total Energy Package (CASTEP) software was used (version 8.0)[3]. This code uses a projector-augmented wave (PAW) method, with plane waves describing valence electrons and pseudopotentials representing core electrons. All calculations used the exchange-correlation functional of Perdew, Burke, and Ernzerhof (PBE)[4], with dispersion corrections specified according to Tkatchenko and Scheffler (TS)[5]. Input files required for these calculations were generated using a Python script, **castepgen.py**, which is included in the data archive. The script makes calls to the program CIF2Cell[6], which in turn generates the .param and .cell

input files required for CASTEP. The main purpose of using this Python script was the ability to loop over all crystal structures (in .cif format) in a given directory. The following (selected) parameter values were used in the .cell files:

KPOINTS_MP_SPACING : 0.05	# specifies a *k*-point spacing of 0.05 Å^{-1}
KPOINTS_MP_OFFSET : 0.25 0.25 0.25	# specifies origin location in fractions of the unit cell
FIX_ALL_CELL : true	# all unit cell parameters are held fixed
FIX_COM : true	# centre of mass is fixed (avoids translation)
FIX_ALL_IONS : false	# enables all atomic positions to be varied

The following (selected) parameter values were used in the input .param files:

xc_functional: PBE	# use of PBE DFT exchange correlation functional
sedc_apply: true	# enables use of a dispersion correction scheme
sedc_scheme : TS	# specified TS dispersion corrections
cut_off_energy: 520 eV	# plane wave kinetic energy cutoff value
geom_disp_tol : 3.0e-003	# geometry displacement tolerance (in Å)
geom_energy_tol : 1.07788e-005	# geometry energy tolerance (in eV atom^{-1})
geom_force_tol : 0.030367	# maximum allowed residual force (in eV Å^{-1})

The geometry energy and geometry force tolerance values are derived from the parameters used by van de Streek et al. in Ref. [7].

Although copies of all .cell and .param files used as inputs are included in the data archive, the files **ABINOS.cell** and **ABINOS.param** are given in the data archive (computational subdirectory) as representative examples of input files. Outputs from the calculations (files having general formats of **abcdef01.castep** and **abcdef01-out.cif**, with **abcdef01** representing the associated CSD refcode) are also contained in the data archive.

After fixed-cell structure relaxations were completed, the pairwise overlay process was repeated on the remaining structure pairs. The protocols associated with this overlay process mirrored those described earlier (the Python script used, **overlay2.py**, is in the data archive), and key information can be found in the Excel spreadsheet in the data archive (**overlay_results_and_DFT_summaries.xlsx**, spreadsheet tab: **structures above 0.25 A**). Structural differences were deemed to be resolved if all atom-atom differences had reduced to below the threshold of 0.25 Å. Of the **658** structure pairs considered at this stage, the structural differences of **521** pairs were resolved, while **134** structure pairs remained significantly different. A further **3** structure pairs (EGUJEV/EGUJEV01; BUYRUI01/BUYRUI02; BUYRUI01/BUYRUI03) diverged to clearly different polymorphic forms (i.e., the overlay process could not be meaningfully performed). As before with the set of structure pairs (i.e., prior to structure relaxations), the number of structure pairs possessing specified amounts of local and overall structural differences are provided (Figure S13), as are the most common types of local differences (Table S5). An additional analysis was performed, concerning how far in space each structure moved in relation to its starting geometry. The information generated, along with the Python script used to generate it (**analyseGOstats.py**), can be found included in the data archive (**fixed_cell_stats.csv**).

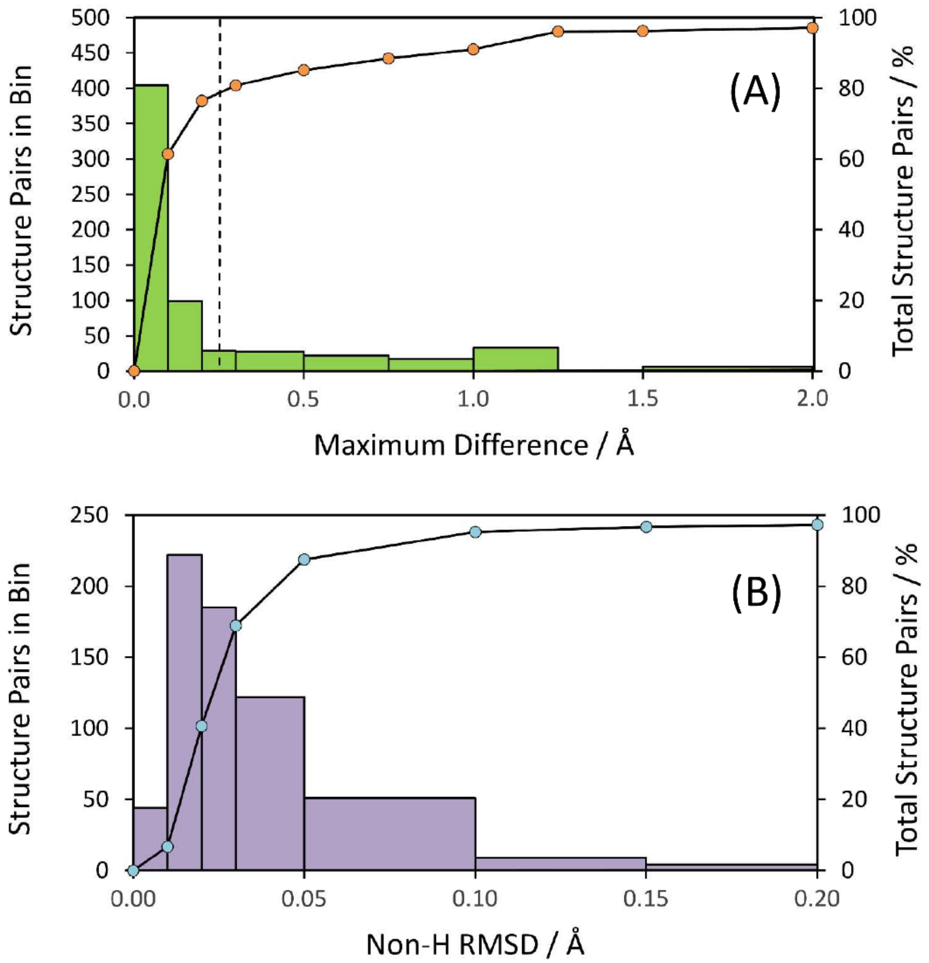

Figure S13. Histograms after performing fixed-cell dispersion-corrected DFT structural optimizations. The primary vertical axis indicates the number of structure pairs possessing (A) a maximum (local) difference, and (B) a heavy-atom (non-H) RMSD within the values specified by the horizontal axis. The secondary vertical axis indicates the cumulative total number of structure pairs, expressed as a percentage of the **655** structure pairs that could be meaningfully compared at that point. The **134** structure pairs having a maximum (local) difference of ≥ 0.25 Å were selected for a further (i.e., non-fixed unit cell) structural relaxation, again using dispersion-corrected DFT. The vertical dashed line in (A) is a guide for the eye.

Table S5. Most common types of structure pair differences after a fixed-cell structure optimization

Difference Type	Number of Pairs	Percentage[a]	Number of Pairs (> 0.25 Å)	Percentage (> 0.25 Å)[b]
Me	240	36.6	88	65.7
CH_2	126	19.2	12[c]	9.0
CH	115	17.6	1	0.7
OH	79	12.1	17	12.7
H_2O	21	3.2	2	1.5
NH_2	15	2.3	1	0.7
CO	15	2.3	0	0.0
NH_3^+	14	2.1	1	0.7
All others	30[d]	4.6	12[e]	9.0

[a] Percentages are with respect to the **655** structure pairs that could be meaningfully compared. As mentioned above, **3** structure pairs diverged structurally to such an extent that they could not be meaningfully overlaid.

[b] Percentages are with respect to the **134** structure pairs (out of a possible **655**), where the maximum local difference was greater than 0.25 Å. These pairs were carried forward to the variable-cell DFT optimizations.

[c] Potentially misleading, as 9 of the 12 pairs of structures in this category corresponded to the same form of a molecule (refcode: HXMTAM22) that happened to have a very large number of alternative structure determinations.

[d] Difference types grouped into the 'All others' category (number of pairs in parentheses) at this point include: NH (8); Cl^- (4); NH_4^+ (3); ring (3); H-transfer (2); MeOH (1); NO_2 (1); CCl (1); NO (1); rotamer (1); ClO_4^- (1); other non-specified differences (4).

[e] Difference types grouped into the 'All others' category (number of pairs in parentheses) at this point include: NH (1); NH_4^+ (3); ring (3); H-transfer (2); CCl (1); rotamer (1); other non-specified differences (1).

To provide greater flexibility for the convergence towards a common structure, the **134** structure pairs that remained significantly different after the fixed-cell structural relaxations (and also the **3** pairs that had diverged to different polymorphs) were subjected to a second structural relaxation in which the unit cell parameters were allowed to vary (i.e., in the .cell input file, the 'FIX_ALL_CELL' parameter was set to 'false'). All input and output files for these calculations are included in the data archive subdirectory **/computational/variable_cell**.

These variable-cell relaxations resolved an additional **20** structure pairs (15% of 134), with **1** structure pair (TETCYH01/TETCYH12) diverging into different polymorphic forms, thus making a total of **4** pairs incomparable at this point). As a result, **113** structure pairs remained significantly distinct. The differences are again concentrated on systems with methyl- or hydroxyl-group differences (Table S6). While there appear to several systems having CH_2 differences, 9 of the 11 structure pairs in this group belong to the same form of a molecule with several alternative structure determinations.

Table S6. Most common types of structure pair differences after both fixed-cell and variable-cell structure optimizations

Difference Type	Number of Pairs (> 0.25 Å)	Percentage (> 0.25 Å)[a]
Me	73	64.6
OH	17	15.0
CH_2[b]	11	9.7
ring	3	2.7
NH_4^+	3	2.7
H-transfer	2	1.8
All others[c]	4	3.5

[b] Percentages are with respect to the **113** structure pairs, out of a possible **134**, where the maximum local difference was > 0.25 Å. As such, these pairs were carried forward to the calculation of NMR parameters.
[b] Potentially misleading, as 9 of the 11 pairs of structures in this category corresponded to the same form of a molecule (refcode: HXMTAM22) that had a very large number of alternative structure determinations.
[c] Difference types grouped into the 'All others' category (number of pairs in parentheses) include: NH_2 (1); NH (1); rotamer (1); other non-specified differences (1).

4. Calculating ^{1}H and ^{13}C Nuclear Magnetic Resonance Spectra for Unreconciled Structure Pairs

For the **113** structure pairs whose differences remained unresolved after the two rounds of dispersion-corrected DFT calculations (plus the **4** pairs that diverged into different polymorphs), the magnetic shielding values at the ^{1}H and ^{13}C nuclei were calculated using the gauge-including PAW (GIPAW) approach as implemented in CASTEP[8]. All input and output files for these calculations are included in the data archive, subdirectory: **/computational/NMR**. Synthetic ^{1}H and ^{13}C NMR spectra were generated from these calculated magnetic shielding values using the Python script **shieldings_compare.py** (included in the data archive), with 0.25 ppm of Lorentzian line broadening being applied in all cases.

Although metrics exist in the literature for distinguishing chemical structures from one another *via* their ^{1}H and ^{13}C NMR spectra[9], these assume a reasonably complete assignment has been performed. Since it was not viable to match the computed shielding values arising from the geometry-optimized alternative structures with different site labels in an automated fashion using currently available tools, comparisons between pairs of calculated NMR spectra were done without assignment using **shieldings_compare.py**. Where required, the three ^{1}H shielding values associated with any methyl group were averaged prior to computing the virtual spectrum. At this point, for each peak associated with one of the structures in a given structure pair, this script determined the smallest distance (in ppm) relative to all the calculated NMR signals of the other structure in the structure pair. More conservative values for spectral differences (in ppm) are used here when compared against those used in the literature with assigned datasets. Specifically, we considered a pair of unassigned ^{1}H NMR spectra as distinguishable from one another if there existed at least one calculated peak position in one spectrum that was at least 0.5 ppm away from every peak in the other calculated ^{1}H NMR spectrum. For the ^{13}C NMR spectral pairs, this value was chosen to be 2.3 ppm. Results of this process were summarized as whisker plots and can be found in the file: **alternative_structures_NMRsummary.xlsx** in the data archive. The numbers of distinguishable structure pairs, sorted according to difference type, are shown in Table S7.

Table S7. Potential to Distinguish Structure Pairs Based on $^{1}H/^{13}C$ NMR Chemical Shifts[a]

Difference Type	Number of Pairs (> 0.25 Å)	^{1}H only	^{13}C only	Both $^{1}H/^{13}C$	Neither $^{1}H/^{13}C$
Me	73	2	15	1	55
OH	17	1	1	12	3
CH_2	11	0	0	9[b]	2
ring	3	0	1	1	1
NH_4^+	3	2	0	0	1
H-transfer	2	0	0	2	0
Divergent[c]	4	0	0	4	0
All others[d]	4	0	1	2	1

[a] A given pair of structures was deemed as potentially distinguishable using ^{1}H chemical shifts if at least one peak in one of their corresponding unassigned ^{1}H spectra was at least 0.5 ppm away from all other peaks associated with the other structure in the structure pair. For ^{13}C, this shift value was taken to be 2.3 ppm.
[b] Potentially misleading, as these 9 pairs of structures all included the same form of a molecule that happened to have a very large number of alternative structure determinations.
[c] Divergent structures could not be overlaid and as such are believed to represent different polymorphic forms.
[d] Difference types grouped into the 'All others' category (number of pairs in parentheses) include: NH_2 (1); NH (1); rotamer (1); other non-specified differences (1).

Based on the above, most structure pairs having Me groups as their principal structural difference would not be expected to be distinguishable using either ^{1}H or ^{13}C NMR experiments at ambient temperature; using low-temperature measurements to "freeze out" the averaging of the methyl resonances can be expected to improve distinguishability. On the other hand, structure pairs having OH groups as their main structural difference are predicted to be regularly distinguishable (distinguishable in 14 out of 17 pairs). As only 17 structure pairs belonged to the OH group, it was deemed worthwhile to manually inspect and assign the ^{1}H and ^{13}C NMR spectra associated with these structure pairs. Subsequently, RMSD calculations were performed (Table S8 below), and using the literature metrics for ^{1}H and ^{13}C, the likelihood of differentiating these pairs was determined (discussed in the main manuscript).

Table S8. ^{1}H and ^{13}C RMSD and Maximum Deviations for Unresolved "OH" Difference Type Pairs[a]

Pair ID	CSD Refcodes	^{1}H RMSD / ppm	^{1}H Max Dev. / ppm	^{13}C RMSD / ppm	^{13}C Max Dev. / ppm
0029	ADGALA01/10	0.878	1.86	2.26	3.62
0732	DAPJUA/01	2.33	6.86	1.78	2.46
0802	DEZJEX/01	0.669	2.30	1.30	2.53
1019	EDENEH/02	0.355	0.95	2.75	8.00
1020	EDENEH01/02	0.356	0.94	2.77	7.88
1034	EHIYEZ/01	2.25	5.95	2.89	7.47
1256	FURSEM01/17	1.60	5.10	1.47	5.02
1695	IPRPOL/03	0.117	0.27	0.599	1.16
1932	KONTIQ01/02	0.159	0.42	1.03	2.61
2439	NUPJOX/01	2.07	9.95	3.13	6.81
2715	PUYYOX/01	0.557	3.61	0.947	3.47
2941	SANYIP/02	3.61	10.16	5.00	10.78
3492	TRDECA01/02	0.336	0.71	0.551	1.19
3844	WOVYUB/05	2.75	11.27	2.88	7.55
3849	WOVYUB04/05	2.72	11.14	2.86	7.56
4006	YIZGOE/01	0.898	3.00	1.05	3.52
4127	ZUHRID/02	0.854	2.13	2.65	6.24

[a] Data result from GIPAW DFT calculations using fully-optimized input structures and assigned spectra. The RMSD data columns are also depicted in **Figure 2** of the main manuscript.

Table S9. Statistics for effects of fixed-cell geometry optimisations as a function of diffraction experiment type

Diffraction experiment	Number of structures	Median maximum atomic displacement[a] / Å	Median RMSD[a] / Å
Single-crystal X-ray	986	0.30	0.042
Powder X-ray	18	0.4	0.08
Non-X-ray (neutron assumed)	50	0.09	0.03

[a] Values given to 2 and 1 significant figures for first and remaining rows respectively, reflecting differing numbers of data points.

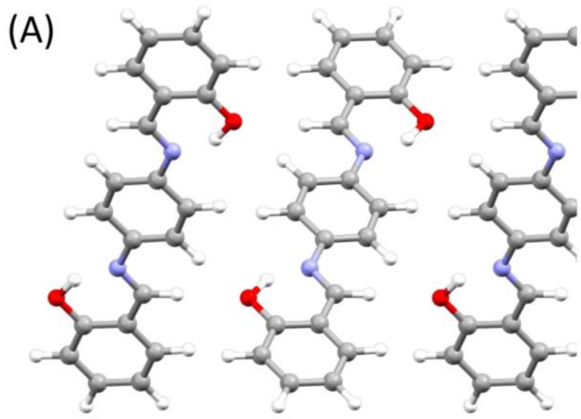

Figure S14. Depiction of the intermolecular packing interactions present in the fully geometry optimized structures of (A) **SANYIP** and (B) **SANYIP02**.

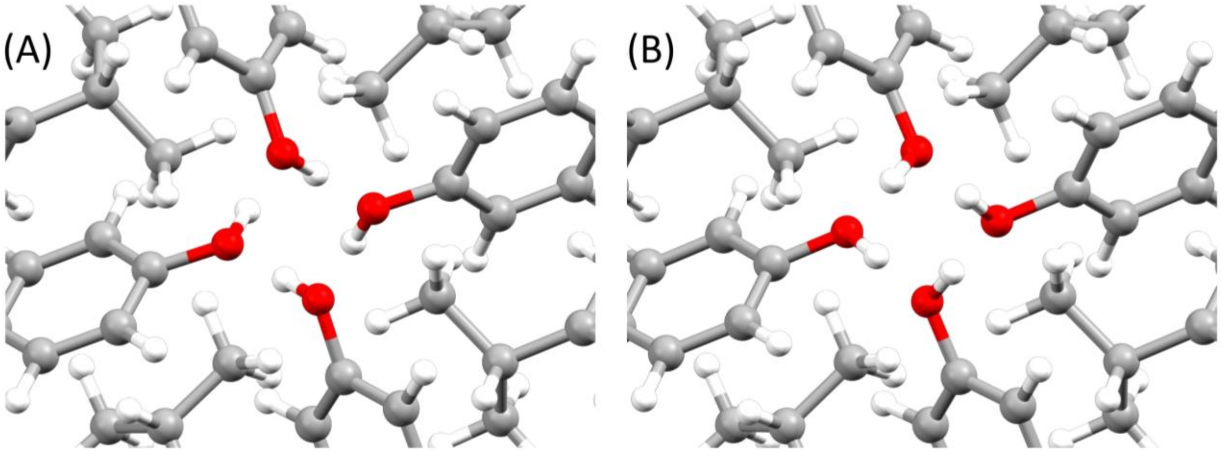

Figure S15. Depiction of the "four-fold symmetry" present in the hydrogen bonding networks of the fully geometry optimized structures of (A) **IPRPOL** and (B) **IPRPOL03**.

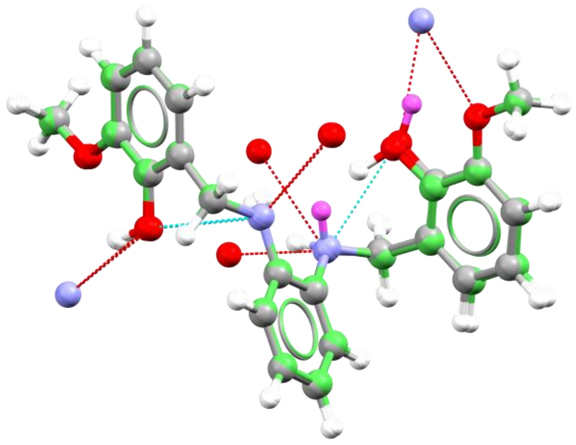

Figure S16. Depiction of the intermolecular interactions present in the fully geometry optimized structures of **EDENEH** and **EDENEH02**. When comparing these two structures the main structural differences are in the

positioning of two hydrogen atoms (one from an NH group and another from an OH group). At these sites of difference, the hydrogen atom for **EDENEH** has been coloured magenta.

References

[1] I. J. Bruno, J. C. Cole, P. R. Edgington, M. Kessler, C. F. Macrae, P. McCabe, J. Pearson, R. Taylor, *Acta Cryst. B* **2002**, *58*, 389-397.

[2] C. F. Macrae, I. J. Bruno, J. A. Chisholm, P. R. Edgington, P. McCabe, E. Pidcock, L. Rodriguez-Monge, R. Taylor, J. van de Streek, P. A. Wood, *J. Appl. Crystallogr.* **2008**, *41*, 466-470.

[3] S. J. Clark, M. D. Segall, C. J. Pickard, P. J. Hasnip, M. I. Probert, K. Refson, M. C. Payne, *Zeitschrift für Kristallographie-Crystalline Materials* **2005**, *220*, 567-570.

[4] J. P. Perdew, K. Burke, M. Ernzerhof, *Phys. Rev. Lett.* **1996**, *77*, 3865.

[5] A. Tkatchenko, M. Scheffler, *Phys. Rev. Lett.* **2009**, *102*, 073005.

[6] T. Björkman, *Comput. Phys. Commun.* **2011**, *182*, 1183-1186.

[7] J. van de Streek, M. A. Neumann, *Acta Cryst. B* **2010**, *66*, 544-558.

[8] a C. J. Pickard, F. Mauri, *Phys. Rev. B* **2001**, *63*, 245101; b J. R. Yates, C. J. Pickard, F. Mauri, *Phys. Rev. B* **2007**, *76*, 024401.

[9] E. Salager, G. M. Day, R. S. Stein, C. J. Pickard, B. Elena, L. Emsley, *J. Am. Chem. Soc.* **2010**, *132*, 2564-2566.
